# Dosimetric characterization of scatter foil‐enhanced contact collimation for small superficial electron beam therapy

**DOI:** 10.1002/acm2.70484

**Published:** 2026-02-09

**Authors:** Abdulaziz Alhussan, Richard Crilly

**Affiliations:** ^1^ Department of Radiation Medicine Oregon Health and Science University Portland Oregon USA; ^2^ Present address: Department of Biochemistry and Molecular Biology University of British Columbia Vancouver British Columbia V6T 1Z4 Canada

**Keywords:** cerrobend, electron beam therapy, isodose distribution, penumbra, scatter foil, skin collimator, small field dosimetry, superficial tumors

## Abstract

**Purpose:**

This study evaluates the dosimetric impact of integrating thin metallic scatter foils with Cerrobend contact skin collimators to improve dose uniformity, conformality, and distal tissue sparing in small superficial electron fields.

**Materials and methods:**

Electron beams of 8, 12, and 15 MeV from an Elekta Versa HD LINAC were delivered through a Cerrobend skin collimator with a 2.0 cm aperture at 100.0 cm SSD. Thin aluminum (Al) and lead (Pb) foils (< 2.50 mm) were placed on the collimator. Gafchromic EBT3 film in a solid‐water phantom was used to measure depth‐dose distributions and isodose profiles following TG‐235–consistent calibration.

**Results:**

Scatter foils produced thickness‐ and Z‐dependent modulation of beam characteristics. At 8 MeV, the thickest Pb foil (1.07 mm) reduced the practical range (*R*_p_) by ∼45% and shifted *D*_m__a_ *_x_* proximally by ∼0.5 cm, yielding substantial distal tissue sparing. Al foils caused smaller *R_p_* reductions (15%–25%) but improved lateral dose uniformity, producing smoother and more symmetric isodose contours. The 90% isodose diameter decreased with foil thickness for both materials, with Pb showing the largest contraction (∼20%–25%, energy‐dependent), enhancing field conformality. Penumbra width increased slightly for thin foils but stabilized or narrowed for larger thicknesses. These effects diminished at 15 MeV, indicating reduced sensitivity of high‐energy electrons to thin‐foil perturbation.

**Conclusions:**

Thin metallic foils placed on Cerrobend skin collimators enable a controllable balance between dose uniformity (improved with Al) and conformality with distal sparing (enhanced with Pb). This simple, LINAC‐independent configuration offers a cost‐effective method for modulating small‐field electron beam characteristics and may serve as a practical adjunct for treating superficial lesions.

## 1 INTRODUCTION

Skin cancer is the most common malignancy in the United States, with non‐melanoma skin cancers accounting for an estimated 5.4 million diagnoses each year, and melanoma representing approximately 5% of all new cancer cases and about 1% of cancer deaths, with both incidence and mortality continuing to rise.^1^, ^2^ Treatment selection depends on tumor type, anatomic site, cosmesis, comorbidities, and available resources, and may include surgery, radiotherapy, chemotherapy, photodynamic therapy, or biologic therapy.^3^ For superficial or near‐surface disease, electron beam therapy is often preferred because of its favorable dosimetric characteristics, including a high surface dose, a broad uniform dose region around the depth of maximum dose, and a steep distal fall‐off that limits irradiation of underlying tissues.^4^ However, when treating small lesions, the electron dose distribution becomes less predictable and less uniform, making it difficult to encompass the target within clinically desirable isodose curves without producing hot or cold spots, thereby compromising both tumor coverage and distal tissue sparing.^5^, ^6^, ^7^ Improving uniformity in small electron fields (smaller than those required for full lateral scatter equilibrium) remains a persistent clinical and dosimetric challenge.

Contact skin collimation using high‐Z materials such as lead or Cerrobend (Z_eff ≈ 84) remains a standard and widely used approach for sharpening the penumbra and limiting dose to distal tissues in small‐field electron therapy.^8^, ^9^, ^10^, ^11^, ^12^ Optimal dosimetric performance is achieved when the collimator is placed directly in contact with the skin; however, this configuration increases surface dose and photon contamination, and the depth‐dose characteristics remain highly dependent on field size.^4^, ^9^, ^12^ Several recent studies have reinforced the importance of surface‐proximal electron collimation and have examined limitations associated with traditional Cerrobend inserts. For example, Herchko et al. demonstrated that patient‐specific 3D‐printed skin collimators can reproduce the dosimetry of commercial cutouts with excellent agreement in field size, output, and surface dose, underscoring ongoing efforts to refine electron beam shaping methods.^12^ Paschal et al. similarly showed that a surface‐conforming electron MLC (SCEM) can achieve sharper penumbrae and reduced out‐of‐field dose compared with Cerrobend, further illustrating the dosimetric benefits of collimation placed near the patient surface.^13^ Additional design strategies, such as beveled collimator edges, have improved field conformity but can compromise lateral dose uniformity for small circular fields.^14^ Moreover, while high‐Z collimators effectively suppress lateral scatter, their higher radiative losses, which scale approximately with *Z* ^2^ and electron energy, can increase bremsstrahlung production.^4^, ^15^ Other work has specifically explored small‐field uniformity enhancement using magnetic and foil‐based techniques. Phaisangittisakul et al. integrated magnetic collimators (up to 2.0 T) with thin metallic foils to modulate electron range and fluence, finding that low‐Z foils such as aluminum (Al) improved depth‐dose uniformity with reduced bremsstrahlung compared with high‐Z foils such as lead (Pb).^16^ Although such systems demonstrated smoother dose profiles, the need for complex magnetic assemblies and precise multi‐component alignment limited their clinical practicality.

**FIGURE 1:**
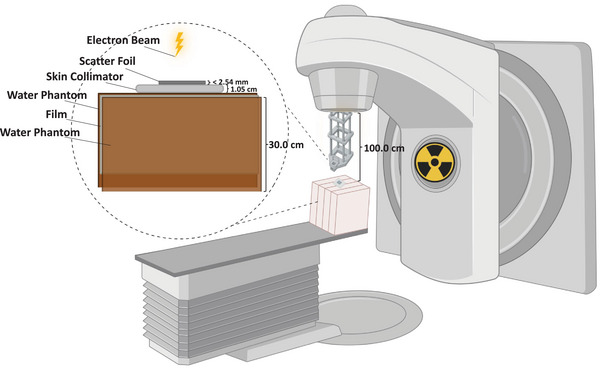
Experimental setup for evaluating small‐field electron beam collimation. Conceptual schematic showing a thin metallic foil (Al or Pb, < 2.5 mm) mounted on a Cerrobend contact skin collimator positioned flush with the phantom surface. Electron beams (8, 12, and 15 MeV) were delivered through a 2 cm circular aperture at a clinical SSD = 100 cm. Gafchromic EBT3 film was placed within a solid‐water phantom for dose measurement. Created with BioRender.com.

To our knowledge, no prior study has evaluated the use of a thin metallic foil integrated with a contact skin collimator to enhance small‐field electron dose uniformity through a simple, clinically feasible, and LINAC‐independent configuration. In this proof‐of‐concept study, we investigated the dosimetric effects of placing low‐ and high‐Z thin metallic foils (Al and Pb, each less than 2.50 mm thick)^16^ on a Cerrobend contact skin collimator for small circular fields of 2.0 cm diameter using 8, 12, and 15 MeV beams at a clinical SSD (Figure 1). Al and Pb were chosen as low‐ and high‐Z extremes to capture the full range of scattering behavior. Pb provides a well‐characterized high‐Z reference, but its toxicity might limit clinical use; in practice, less toxic high‐Z materials such as tungsten or brass may serve as safer alternatives.^17^ This approach is hypothesized to locally emulate aspects of head‐scatter broadening while the skin‐collimator aperture focuses electrons into the treatment area, thereby improving dose uniformity and tissue sparing for small superficial targets without requiring LINAC modification or specialized hardware.

## 2 MATERIALS & METHODS

### 2.1 Skin collimators and field design

A 6 × 6 cm^2^ cutout was mounted in the electron applicator, and the field was further collimated using in‐house–fabricated Cerrobend surface collimators positioned directly on the phantom surface. Each skin collimator had a 2.0 cm circular aperture (representing a small electron field) and a square body extending beyond the treatment area to prevent scattered electron contamination. The Cerrobend collimators were cast from melted alloy (50% bismuth, 26.7% lead, 13.3% tin, and 10% cadmium) poured into custom molds of the desired geometry. Collimator thickness ensured ≤5% transmission based on standard Cerrobend attenuation equations. For Pb, the required thickness could be given by: $t_{\mathrm{Pb}}\;\left(\mathrm{mm}\right)=0.5E_{0}\left(\mathrm{MeV}\right)+1$, and for Cerrobend, the thickness is given by: $t_{c}\;\left(\mathrm{mm}\right)=1.2t_{\mathrm{Pb}}\left(\mathrm{mm}\right)$.^4^, ^10^, ^15^

### 2.2 Scatter foil materials

**FIGURE 2:**
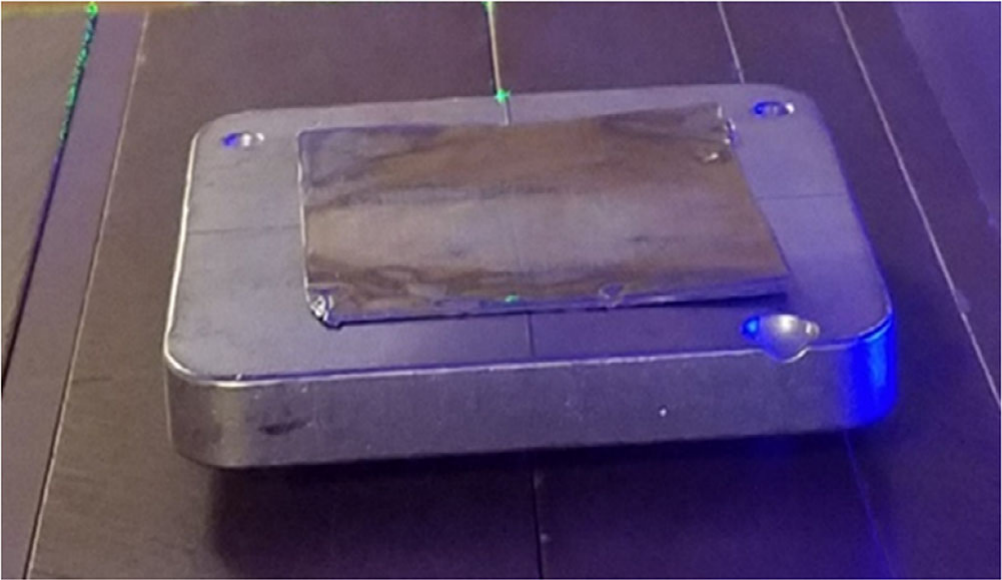
Cerrobend skin collimator used in this experiment with Pb foil on top.

Flat metallic foils of Pb (*Z* = 82; 0.152 mm thick each) were obtained from Nuclear Associates (Carle Place, NY, USA; now Fluke Biomedical), and Al (*Z* = 13; 0.635 mm thick each) from Rose Metal Products, Inc. (Springfield, MO, USA). Multiple foils were stacked to achieve total thickness up to 1.07 mm for Pb and 2.54 mm for Al. Care was taken to ensure firm contact between layers and to avoid air gaps that might affect scattering and attenuation. These materials were selected to represent high‐ and low‐Z extremes, providing a broad range of scattering and attenuation characteristics for evaluating dose uniformity. Each foil was placed directly on top of the Cerrobend skin collimator, fully covering the open aperture. This configuration is shown in Figure 2.

### 2.3 Film dosimetry and calibration

Gafchromic EBT3 film was used for relative dosimetry because of its high spatial resolution, near tissue equivalence, and minimal energy dependence in the therapeutic electron range.^18^ Films were handled under consistent ambient conditions, oriented uniformly, and stored in light‐protective envelopes for at least 24 h post‐irradiation to allow for full polymerization. Calibration films were irradiated using a 10 × 10 cm^2^ open field at 100.0 cm SSD with doses ranging from 0–800 cGy in increments of 50–100 cGy, following the AAPM TG‐55 film calibration protocol.^19^ Although TG‐55 was historically used for film calibration, the procedures applied in this study remain fully consistent with the updated AAPM TG‐235 recommendations, which confirm the suitability of Gafchromic EBT3 film for relative electron‐beam dosimetry and support the validity of the measurements presented here.^20^ Horizontal calibration films were placed at *D*_max_ for each energy (2.2, 2.8, and 3.4 cm for 8, 12, and 15 MeV, respectively). Vertical calibration films were placed edge‐on between the two phantom halves, aligned such that the uncut edge matched the phantom surface, to verify depth‐dose accuracy and minimize edge artifacts.

### 2.4 Experimental setup

**FIGURE 3:**
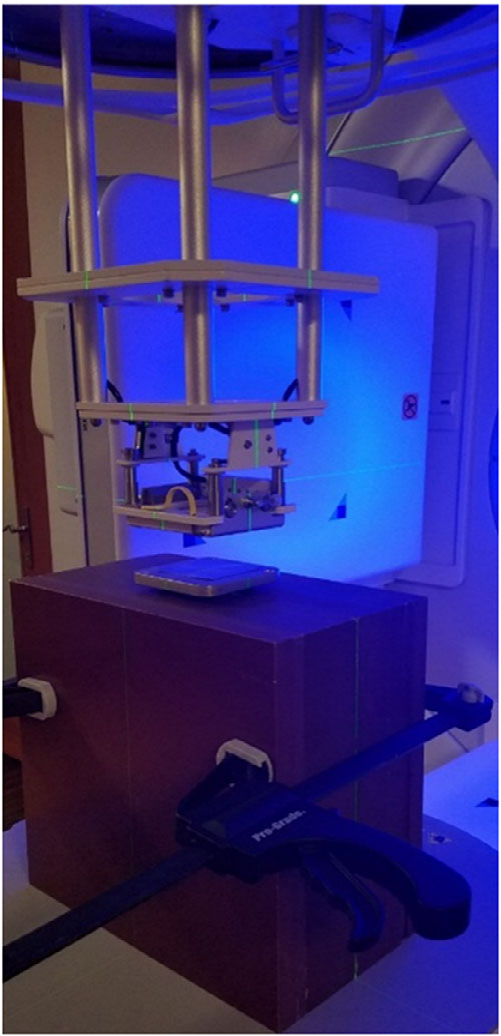
Experimental setup for vertical film irradiation. The Gafchromic EBT3 film was positioned at midplane within the solid‐water phantom, with the Cerrobend contact skin collimator and metallic scatter foil (Al or Pb) placed directly on the phantom surface.

All experiments were conducted using an Elekta Versa HD LINAC (Stockholm, Sweden). Electron beams of 8, 12, and 15 MeV were delivered at an SSD of 100.0 cm through a 6 × 6 cm^2^ cutout mounted in the electron applicator to represent a realistic small‐field configuration. A Nomos solid‐water phantom (Gammex/Sun Nuclear, Melbourne, Florida, USA) was used as a tissue‐equivalent medium due to its close match to water in electron density. The phantom measured 30 × 30 × 18 cm^3^ when assembled, with individual slabs of 0.2–6.0 cm thickness (Figure 3). For all measurements, film strips (5 × 10 cm^2^ to 5 × 12 cm^2^) were sandwiched horizontally at the midplane between two 9.0 cm halves of the phantom, aligned perpendicular to the beam. Thicker slabs were positioned internally to correct for backscatter and ensure uniform dose. A 2.0 cm Cerrobend skin collimator with Pb or Al scatter foils of different thicknesses was centered on the beam axis. All measurements were performed at 600 MUs to achieve sufficient film response while minimizing scanner saturation. A control condition (Cerrobend only, no foil) was included for each energy.

### 2.5 Film scanning and image analysis

All films were scanned using an Epson Expression 10000XL flatbed scanner (Seiko Epson Corporation, Suwa, Nagano, Japan) at 72 dpi with consistent orientation (face down, landscape mode). Scans were processed in DoseLab software (Mobius Medical Systems, TX, USA). Unexposed film images were used for 2D uniformity correction. Calibration curves (OD vs. dose) were generated for each energy using third‐order polynomial fitting. DoseLab's region‐of‐interest (ROI) tool was applied to convert OD images into dose maps, which were then analyzed for PDD, lateral profiles, isodose curves, and all other relevant data.

### 2.6 Statistical analysis

Each film measurement was repeated three times for reproducibility, and results are reported as mean ± 1 standard deviation. Statistical comparisons between groups (foil materials, beam energies, or foil thicknesses) were performed using Welch's *t*‐test, which accounts for unequal variances and small sample size. PDD curves were normalized to their own maximum value on the central axis (100%) unless otherwise noted. Quantitative parameters extracted included: *D*_max_, 90% isodose diameter, practical range (*R*_p_), and penumbra width.

## 3 RESULTS

### 3.1 Isodose curves

Representative isodose maps for 8 MeV, 12 MeV and 15 MeV are shown in Figure 4. Without a scatter foil, the 90% isodose region was broad but laterally non‐uniform near the collimator edge. Introducing Al foils produced smoother, symmetric contours that gradually contracted with increasing thickness (0.64–2.54 mm), indicating mild field narrowing while maintaining dose uniformity. Pb foils yielded stronger attenuation and scattering, shifting dose regions toward the surface and reducing lateral spread. The 10%–50% contours became tightly spaced, demonstrating a sharper gradient and improved distal tissue sparing. Similar qualitative behavior was observed for higher energies, although the magnitude of the effect decreased with beam energy.

FIGURE 4Isodose curves (10%, 25%, 50%, 80%, and 90%) for 8, 12, and 15 MeV electron beams at 600 MU, shown for the no‐foil condition and for all Al and Pb foil thicknesses. Each panel uses identical spatial scaling to allow direct comparison of field narrowing, dose uniformity, and distal‐tissue sparing across energies and materials.

**Figure:**
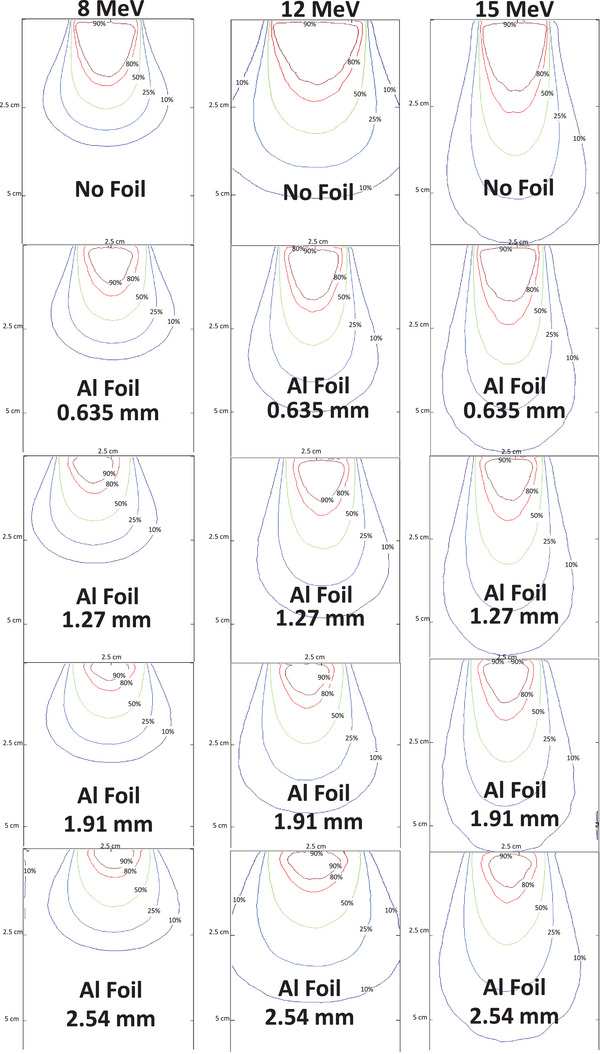

**Figure:**
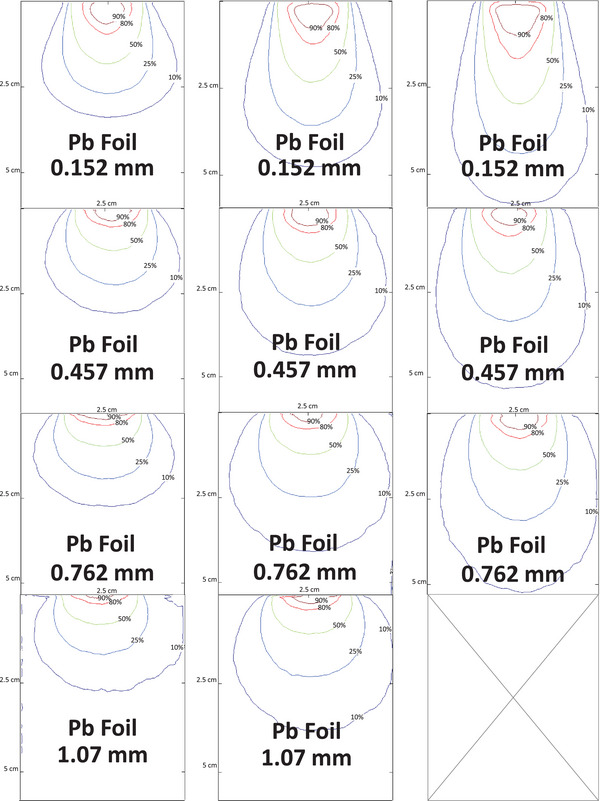

### 3.2 Depth dose distributions

Central‐axis PDD curves for 8, 12, and 15 MeV are presented in Figures S1–S3, respectively. Both *R*_p_ and *D*_max_ decreased as foil thickness increased, with Pb showing the steepest attenuation. At 8 MeV, a 1.07 mm Pb foil shortened *R*_p_ by roughly 45% and moved *D*_max_ proximally by ≈ 0.5 cm relative to the no‐foil case. Al foils produced smaller reductions (≈ 15%–25%), confirming that high‐Z foils degrade electron energy more strongly through enhanced collisional and radiative losses.^4^ At 15 MeV, these differences diminished, indicating reduced sensitivity of higher‐energy beams to thin‐foil scattering.

### 3.3 Quantitative beam parameters

**FIGURE 5:**
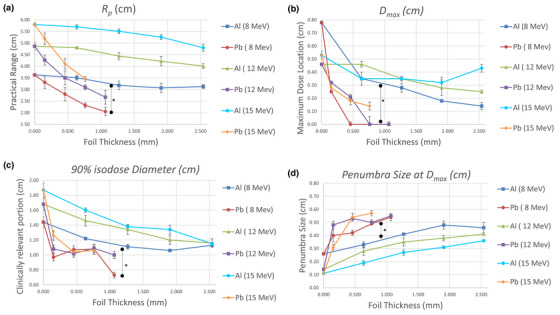
(a) *R*_p_, (b) *D*_max_, (c) 90% isodose diameter, and (d) Penumbra size at *D*_max_, for Pb & Al scatter foils at 8, 12 & 15 MeV. * *p < 0.05*.

Figure 5 summarizes the extracted metrics for all energies. Practical range $R_{p}\;$(Figure 5a) and *D*_max_ depth (Figure 5b) decreased steadily with increasing foil thickness, particularly for Pb. The 90% isodose diameter (Figure 5c) decreased accordingly, demonstrating lateral field narrowing. Penumbra width at *D*_max_ (Figure 5d) showed a non‐linear pattern, slight widening for thin foils followed by stabilization or narrowing at greater thicknesses, reflecting competing effects of angular scatter and beam hardening. Statistically significant differences (*p < *0.05*) were observed between Pb and Al at all energies.

### 3.4 Penumbra size versus depth

To further evaluate lateral fall‐off behavior, Figure S4 plots penumbra size at the 90%, 50%, and 25% isodose levels ($D_{90}$, $D_{50}$, $D_{25}$). Across all energies, penumbra width increased gradually from $D_{90}$ to $D_{25}$, consistent with beam broadening at greater depths. Pb foils generally exhibited smaller penumbrae at $D_{90}$, confirming sharper high‐dose boundaries, while Al foils maintained broader penumbras at all depths, indicative of smoother but less confined fields. At lower energies, Pb's advantage in steep dose fall‐off was more pronounced, whereas at 15 MeV, differences between materials became minimal.

## 4 DISCUSSION

### 4.1 Practical range ($R_{p}$)

*R* ^p^ represents the maximum penetration depth of electrons before they come to rest as they lose all kinetic energy through collisional and radiative interactions.^4^ Clinically, $R_{p}$ marks the end of the electron dose, beyond which only the bremsstrahlung component contributes to residual dose.^10^ According to standard estimation, the range in water (cm) approximates $0.5\;E_{0}$ (MeV), but the measured $R_{p}$ values in this study were slightly shorter than this theoretical prediction for all “no foil” conditions (Figures 5a, S1–S3).^4^ This deviation likely reflects the smaller field size (2 cm) and absence of full lateral scatter equilibrium, conditions that tend to reduce the effective range. The data, however, demonstrate that $R_{p}$ increases with beam energy and decreases with foil thickness, as expected. For Pb, this decrease in *R*_p_ is near‐linear over the investigated thickness range, whereas for Al, particularly at 8 MeV, the slope decreases at larger thicknesses, producing an apparent saturation rather than a strictly linear trend. In this lowest‐energy and very small field (2 cm), full lateral scatter equilibrium is not established, and thin low‐Z Al foils can introduce enough additional angular spread to modestly increase central‐axis scatter at depth. This effect can partially counter the expected decrease in $R_{p}$ for the first few foil layers. Once more Al stacks are used, the cumulative material interaction becomes sufficient to consistently shift the depth fall‐off proximally, and the expected reduction in $R_{p}$ is again observed. The reduction in *R*_p_ with increasing foil thickness is substantially steeper for Pb, reflecting its higher density (11.34 g/cm^3^) and Z‐number (82) compared to Al (2.7 g/cm^3^ and 13, respectively).^10^ Consequently, Pb introduced greater energy loss and angular scatter per unit thickness, resulting in shorter electron ranges. This behavior agrees with known scattering theory, as the energy loss per unit path length and multiple Coulomb scattering angle both scale with Z.^21^

### 4.2 The depth of maximum dose ($D_{\max}$)

The shift in $D_{\max}$ followed a similar pattern (Figure 5b), moving closer to the surface as foil thickness increased. This shift occurs because a portion of the beam energy is expended within the foil itself, leaving less energy to penetrate into the phantom. The effect was most pronounced for Pb at lower energies (8 MeV), where $D_{\max}\;$ approached the surface. The measured $D_{\max}\;$ positions for higher energies (12–15 MeV) were deeper, as higher‐energy electrons undergo less angular deviation and lose energy more slowly per unit depth.^4^ For the 15 MeV beam, the slight increase in $D_{\max}\;$ observed for the 2.54‐mm Al foil can be explained by high‐energy electron transport physics. At 15 MeV, electrons experience relatively small fractional energy loss when passing through a low‐Z material such as Al, while undergoing substantial small‐angle multiple Coulomb scattering.^4^ This scattering redistributes a portion of the fluence into more oblique trajectories that traverse longer geometric paths in the phantom, thus shifting the depth of peak dose deeper. Additionally, increased δ‐electron production in thicker Al layers broadens the build‐up region and contributes to a deeper $D_{\max}$. ^4^ Because attenuation in Al is weak at high energies, these scattering‐driven mechanisms dominate over range reduction, producing the upward shift in $D_{\max}$ at the largest Al thickness.

### 4.3 The diameter at 90% isodose curve

The 90% isodose diameter is of clinical importance because it typically represents the region in which prescribed doses are delivered.^4^ In this work, the 90% isodose region decreased progressively with increasing foil thickness (Figure 5c). Pb foils consistently yielded smaller 90% isodose diameters compared to Al at equivalent energies and thicknesses, consistent with their higher scattering power and energy‐attenuation characteristics. The isodose plots (Figure 4) visually confirm this contraction of the high‐dose region, with Pb producing steeper dose falloffs and more confined treatment fields. Al, in contrast, maintained broader 90% isodose contours with smoother gradients. The clinically relevant implication is that Pb improves conformality but at the cost of reduced uniformity. The magnitude of distal sparing observed with Pb foils, such as the ∼45% reduction in *R*_p_ at 8 MeV and the visibly tighter 10%–50% isodose spacing, is consistent with expected increases in collisional stopping power and angular scatter for high‐Z materials.^16^ Pb therefore produced the strongest proximal shift in dose deposition, whereas Al yielded more modest reductions, in agreement with their relative attenuation characteristics.

### 4.4 Penumbra behavior at different depths

Penumbra behavior is another critical consideration, as it defines the steepness of the dose gradient at field edges and influences both target coverage and distal tissue sparing.^4^, ^10^ The penumbra width, typically defined between 20% and 80% isodose lines at a reference depth, consists of geometric, transmission, and scatter components.^10^, ^21^ Figures 5d and S4 show penumbra widths at $D_{\max}$, $D_{90}$, $D_{50}$, and $D_{25}$ for the various configurations. At $D_{\max}$, Pb foils generally produced broader penumbras than Al, particularly for thicker foils, reflecting increased lateral scatter near the surface. However, at $D_{90}$, the 8 and 12 MeV beams exhibited a slight decrease in penumbra size with foil thickness regardless of material, while 15 MeV beams showed the opposite trend: gradual penumbra widening with thickness. At mid‐depth ($D_{50}$), penumbra widths converged across all conditions, indicating that the dominant factor at these depths is beam energy rather than foil composition. At deeper depths ($D_{25}$), penumbras increased slightly with both foil thickness and energy, consistent with the cumulative broadening effect of multiple scattering in the medium.^21^ Overall, Al foils tended to produce marginally larger penumbras with thickness, while Pb showed an initial increase followed by stabilization beyond 0.76 mm, likely corresponding to saturation in lateral scatter contribution.^21^ It is important to note that for Pb at 12 MeV, the penumbra width exhibited a modest fluctuation with increasing foil thickness (Figure 5d). We infer that this happened because at this intermediate energy, two competing mechanisms influence the lateral dose fall‐off. Thin Pb foils introduce substantial multiple Coulomb scattering that initially broadens the penumbra, whereas additional foil thickness preferentially absorbs lower‐energy electrons, effectively hardening the beam and slightly reducing the lateral spread.^4^ Once the lower‐energy component is sufficiently attenuated, increased scatter again dominates, leading to stabilization of the penumbra at higher thicknesses. Because this interpretation is based on physical reasoning rather than direct measurements, additional experiments are needed to confirm it.

### 4.5 Overall trends and key findings

Across all energies, consistent modulation patterns were observed, demonstrating that thin metallic foils can tune small‐field electron beam characteristics. Lower‐energy beams (8–12 MeV) exhibited the strongest sensitivity to foil composition and thickness, whereas 15 MeV showed comparatively attenuated effects. Increasing foil thickness reduced $R_{p}$, shifted $D_{\max}\;$ proximally, and contracted the 90% isodose diameter in a material‐dependent manner. Pb foils produced steeper lateral and depth gradients, yielding higher conformality and stronger distal tissue sparing, while Al foils generated smoother dose profiles with improved uniformity and broader lateral spread. These trends confirm that the Cerrobend–foil configuration offers a controllable balance between uniformity, conformality, and sparing, each favored by different foil choices. Pb foils produced the strongest enhancement in distal tissue sparing for example, at 8 MeV the 1.07‐mm Pb foil reduced the practical range $R_{p}$ by approximately 45%, while at 12 MeV reductions remained 30%–35%. Al foils produced more moderate but still clinically meaningful reductions of approximately 15%–25% depending on energy and thickness. Conformality also improved markedly, the 90% isodose diameter contracted by as much as 35%–40% for Pb and 20%–25% for Al in the lower‐energy beams. Al further improved dose uniformity by smoothing high‐dose contours and limiting penumbra widening to ≈ 0.1–0.2 cm, in contrast to Pb, which sharpened field edges but created steeper gradients. These results quantify how foil selection enables deliberate prioritization of either uniformity (Al) or conformality and distal sparing (Pb) depending on clinical objectives. The qualitative isodose maps in Figure 4 reinforce these quantitative differences. Pb produced sharply confined 90% fields with tightly spaced distal contours, desirable for sparing tissue beyond superficial targets. Al maintained broader and more symmetric dose regions with reduced hot‐spot formation, improving coverage uniformity for small lesions.

These findings align with earlier observations by Phaisangittisakul et al. that high‐Z foils primarily enhance attenuation and gradient steepness, whereas low‐Z foils improve uniformity through broader scattering behavior.^16^ The present work extends previous studies by demonstrating that similar modulation can be achieved in a simple, LINAC‐independent configuration integrating a standard skin‐contact Cerrobend collimator with thin external foils. Although dosimetrically effective, practical considerations remain important. Cerrobend contact collimators are heavy and rigid, limiting use on highly curved or mobile anatomical regions where reproducible contact cannot be maintained. In such cases, flexible or additive‐manufactured alternatives may be needed. Thus, the technique is best suited to flatter anatomical surfaces where stable, repeatable skin contact can be achieved across fractions. Although this study used Cerrobend as the skin‐contact collimator, the underlying physical mechanisms driving the observed modulation, electron energy loss, multiple Coulomb scattering, and changes in lateral equilibrium introduced by the external Al and Pb foils, are broadly material independent. As such, the qualitative behaviors we report (foil‐dependent reductions in $R_{p}$, shifts in $D_{\max}$, and changes in field conformality and uniformity) are expected to apply similarly to other contact collimators such as brass, stainless steel, tungsten‐silicone composites, or 3D‐printed tungsten‐filled inserts. Only the exact numerical magnitudes may differ depending on the *Z* and density of the base collimator. This supports the generalizability of our findings to a wider range of clinically used skin‐contact electron collimators.

### 4.6 Study limitations and future work

Although meticulous care was taken during all stages of experimentation, potential sources of uncertainty may have influenced the results. These can be broadly divided into setup‐related and analysis‐related factors. Setup‐related uncertainties include film cutting and handling, alignment of the film within the solid‐water phantom, positioning of the phantom relative to the radiation field, and the alignment of both the Cerrobend collimator and metallic foils with respect to each other and the beam axis. Minor misalignments or air gaps between stacked foils may have introduced variations in scatter conditions, particularly in configurations involving multiple thin layers. Analysis‐related uncertainties primarily stem from film scanning and dose conversion. Variations in scanner settings, film orientation, and ROI selection could contribute to differences in measured optical density and derived dose values. Manual extraction of parameters such as penumbra widths and isodose diameters introduces additional measurement subjectivity. The setup uncertainties observed in this study are comparable in magnitude to those encountered during daily patient setup for small superficial electron fields. As with bolus‐based treatments, perfect reproducibility across fractions is rarely achievable in clinical practice.^22^ In many cases, small day‐to‐day deviations tend to average out over the course of treatment; however, persistent misalignment or inconsistent contact could introduce cumulative errors affecting field shape or surface dose. These practical considerations indicate that, if implemented clinically, foil‐enhanced contact collimation would require careful verification of collimator–skin apposition at each fraction, similar to the vigilance required when treating with bolus or custom cutouts. It is also important to note that, although the overall trends are consistent, specific energy–material combinations exhibited departures from monotonic behavior (e.g., Al at 8 MeV for *R_p_*, Al at 15 MeV for *D_m__a__x_*, Pb at 8 MeV for the 90% isodose diameter, and Pb at 12 MeV for penumbra), underscoring that foil‐based modulation in small fields can produce non‐intuitive dosimetric responses and should be implemented cautiously without full 3D dose verification.

An additional limitation is the absence of treatment‐planning system (TPS) modelling in the present work. For hand‐calculation workflows, scatter foils may require adjusted *R*_p_ *, D*_90_, or output values to account for increased attenuation and angular scatter. Modern TPS platforms equipped with type‐C electron algorithms (e.g., eMC) could approximate the foil by modeling a thin high‐density surface slab with an assigned relative electron density, similar to approaches used for bolus. Although TPS‐to‐film comparison was not feasible under the restricted experimental conditions during the COVID‐19 period, incorporating such validation will be an essential step in future studies to ensure accurate clinical implementation. Future work should reproduce the experiments under better controlled conditions and increased repetitions to improve precision. Further refinement of foil geometry, layering, and thickness could achieve tailored dose modulation for lesions of varying depths and shapes. Combining materials of both low‐ and high‐Z may help balance the smoother dose uniformity characteristic of low‐Z materials with the sharper field conformality provided by high‐Z materials. This could improve the clinical versatility of this technique. Future studies should also extend this investigation to lower electron energies (e.g., 4 and 6 MeV), which are commonly used clinically for very superficial lesions, to assess whether the foil‐dependent trends and non‐monotonic behaviors observed here persist under even stronger lateral disequilibrium conditions.

## 5 CONCLUSIONS

This proof‐of‐concept study demonstrates that integrating thin metallic scatter foils with contact skin collimators offers a simple, cost‐effective, and LINAC‐independent approach for modulating small‐field electron beam characteristics. We evaluated low‐Z (Al) and high‐Z (Pb) foils across multiple energies and thicknesses, we showed that foil composition and thickness significantly influence practical range, depth of maximum dose, field conformality, and penumbra behavior. Pb foils produced steeper dose gradients and sharper field confinement, enhancing conformality and distal tissue sparing, while Al foils yielded smoother, more uniform dose distributions with broader lateral spread. These effects diminished at higher energies, suggesting reduced sensitivity of energetic electron beams to thin‐foil perturbations. These findings confirm that this technique enables controllable tuning between uniformity and conformality, addressing a persistent limitation in small‐field electron therapy. With further optimization, this method has potential to evolve into a clinically adaptable adjunct for treating superficial lesions with improved precision and reproducibility.

## AUTHOR CONTRIBUTIONS

*Conceptualization*: Richard Crilly. *Data curation*: Abdulaziz Alhussan. *Formal analysis*: Abdulaziz Alhussan. *Investigation*: Abdulaziz Alhussan. and Richard Crilly. *Methodology*: Abdulaziz Alhussan and Richard Crilly. *Resources*: Richard Crilly. *Supervision and project administration*: Richard Crilly. *Visualization*: Abdulaziz Alhussan. *Writing—original draft preparation*: Abdulaziz Alhussan. *Writing—review and editing*: Abdulaziz Alhussan and Richard Crilly. All authors have read and agreed to the published version of the manuscript.

## CONFLICT OF INTEREST STATEMENT

The authors declare no conflict of interest.

## Supporting information




**Figure S1**: PDDs for 8 MeV at 600 MUs. (A) no foil, (B) Al foil, (C) Pb foil.

**Figure S2**: PDDs for 12 MeV at 600 MUs. (A) no foil, (B) Al foil, (C) Pb foil.

**Figure S3**: PDDs for 15 MeV at 600 MUs. (A) no foil, (B) Al foil, (C) Pb foil.

**Figure S4**: Penumbra size at (A) D90 (cm), (B) D50 (cm), (C) D25 (cm) of Pb & Al scatter foils for 8, 12 & 15 MeV electron beams at 600 MUs.
