# Supplementary figures and images for: Dosimetric characterization of scatter foil‐enhanced contact collimation for small superficial electron beam therapy
Source: J Appl Clin Med Phys. 2026 Feb 9;27(2):e70484. doi: 10.1002/acm2.70484 (PMC12884950; doi:10.1002/acm2.70484)

**(A)**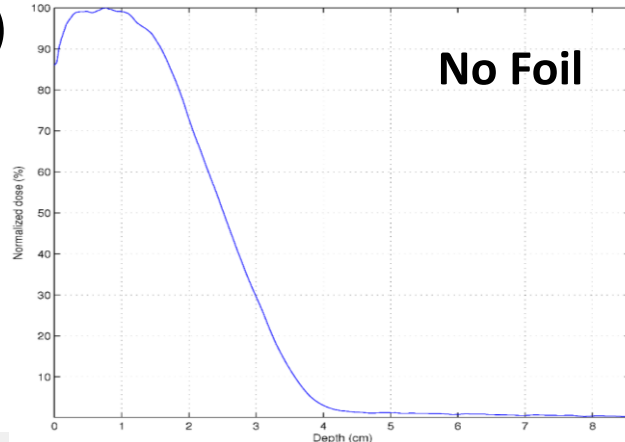**(B)**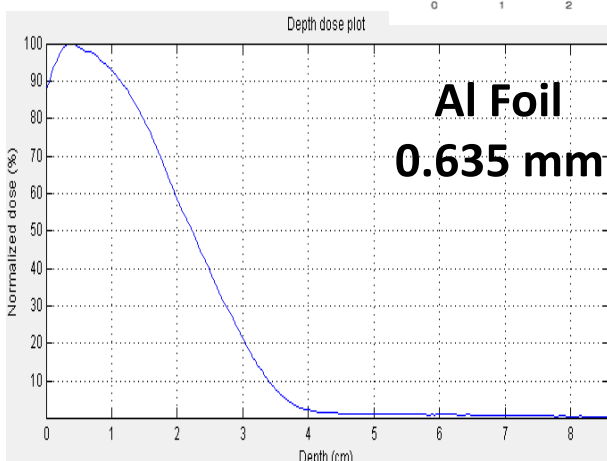**(C)**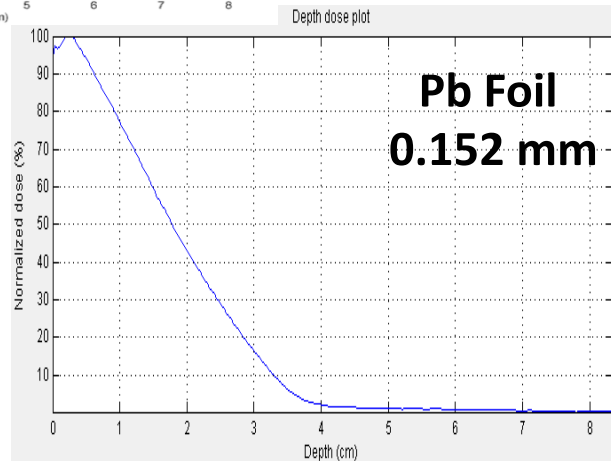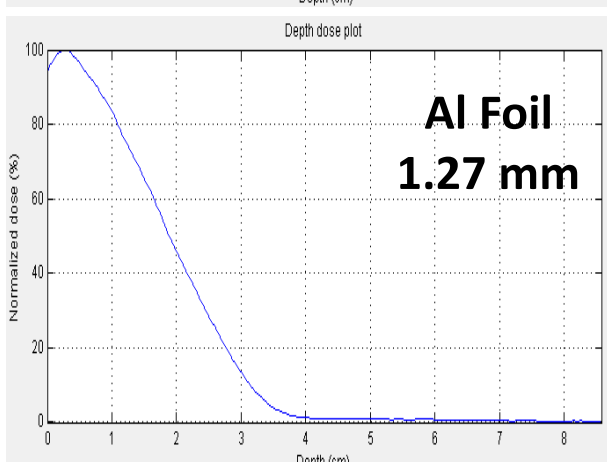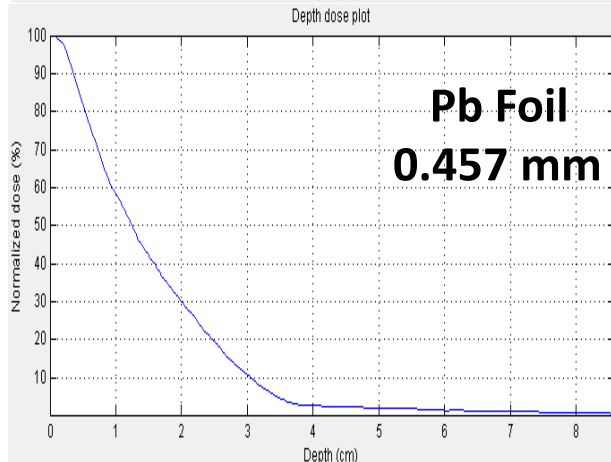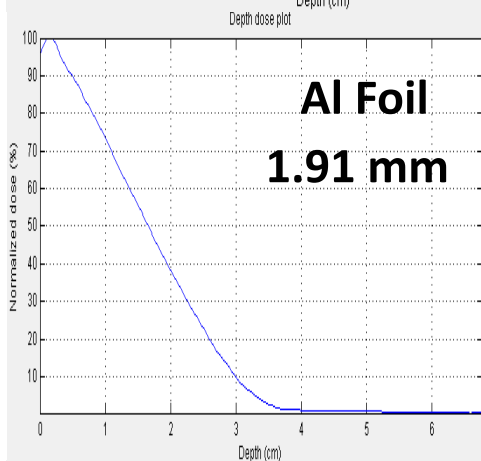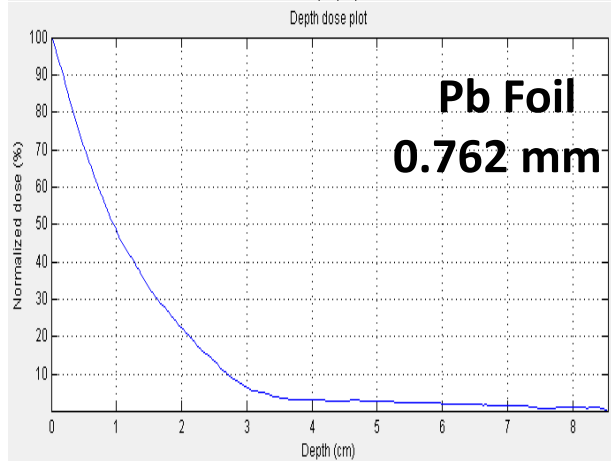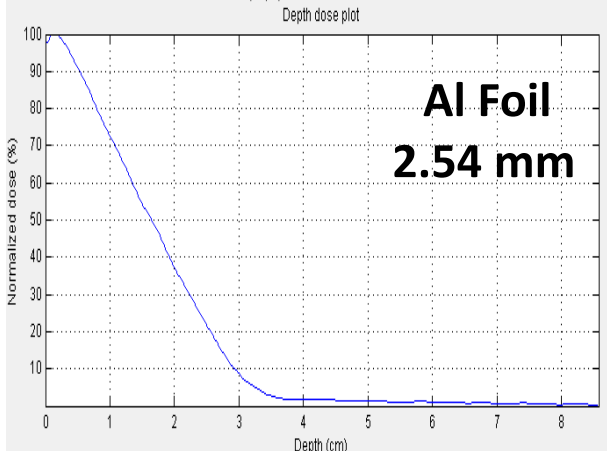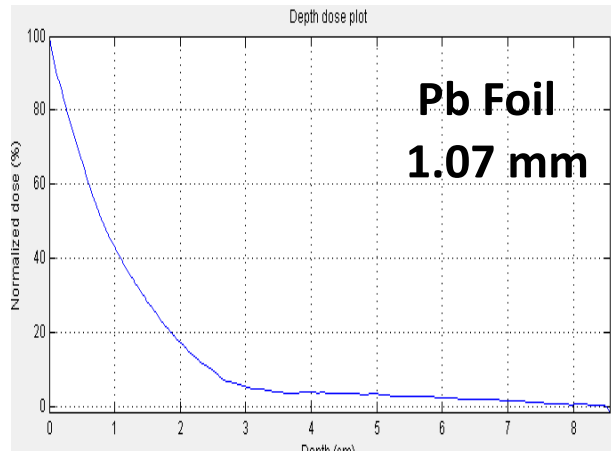

Increasing Foil Thickness

Supplement: Supplementary file 1 — Figure S1: PDDs for 8 MeV at 600 MUs. (A) no foil, (B) Al foil, (C) Pb foil. [file ACM2-27-e70484-s003.pdf]

**(A)**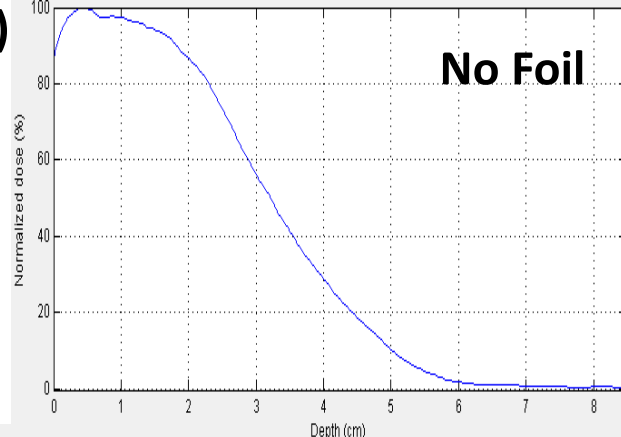**(B)**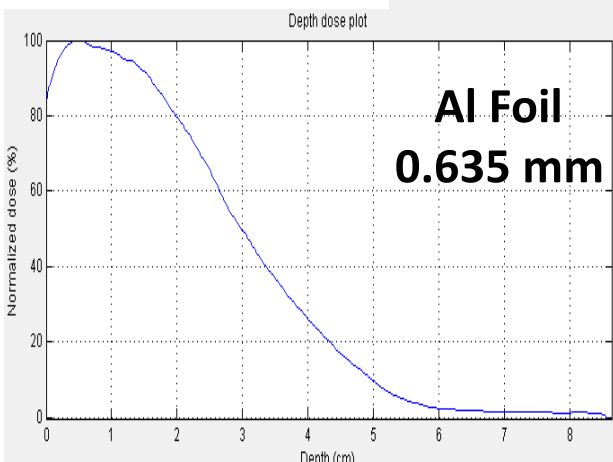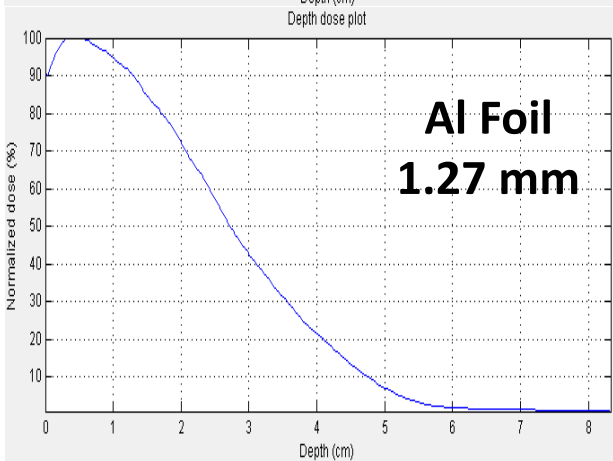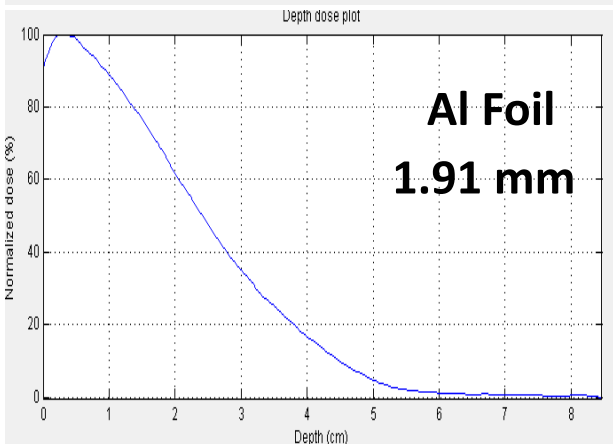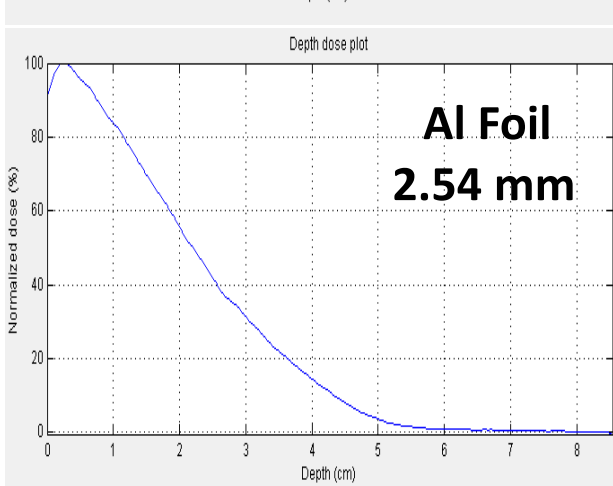**(C)**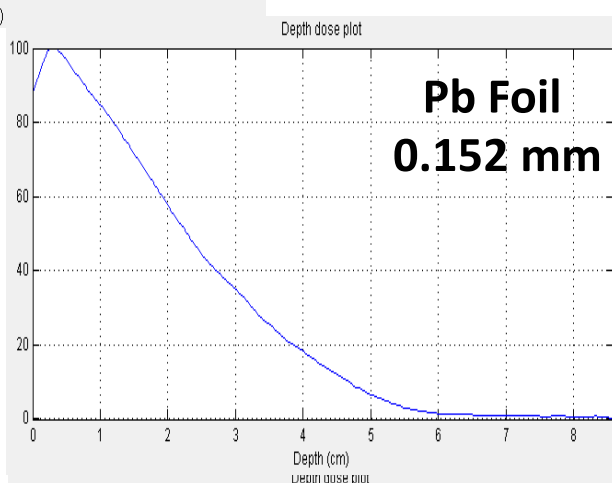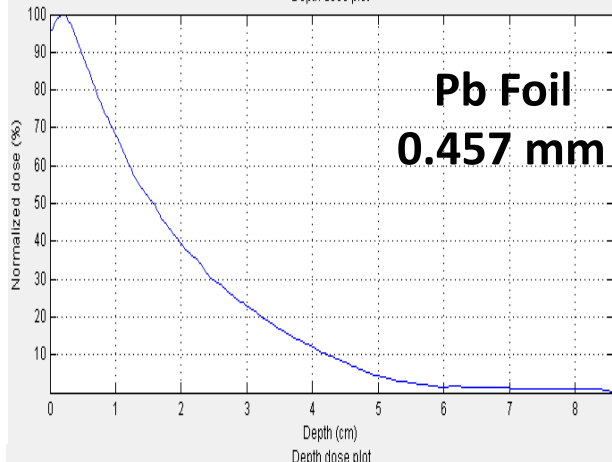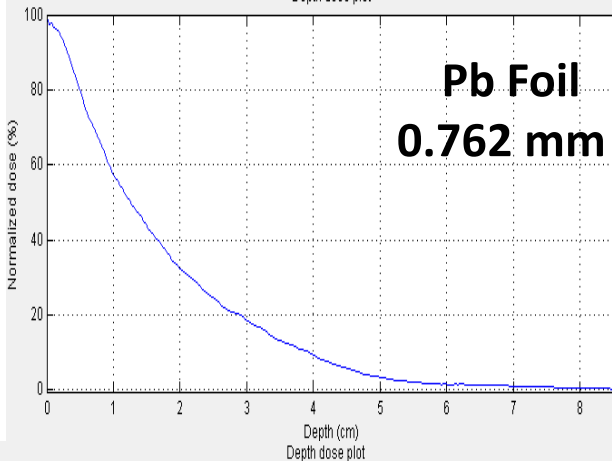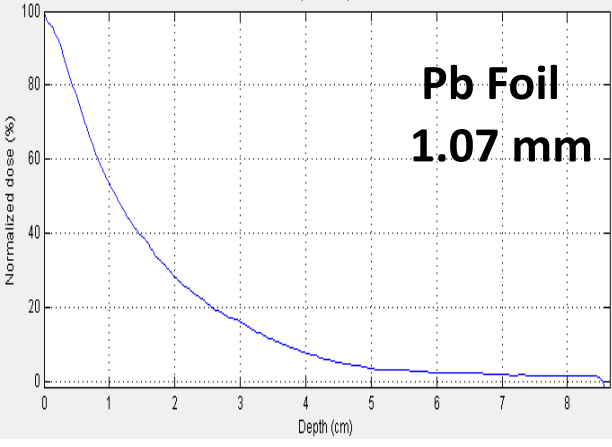

Increasing Foil Thickness

Supplement: Supplementary file 2 — Figure S2: PDDs for 12 MeV at 600 MUs. (A) no foil, (B) Al foil, (C) Pb foil. [file ACM2-27-e70484-s002.pdf]

**(A)**

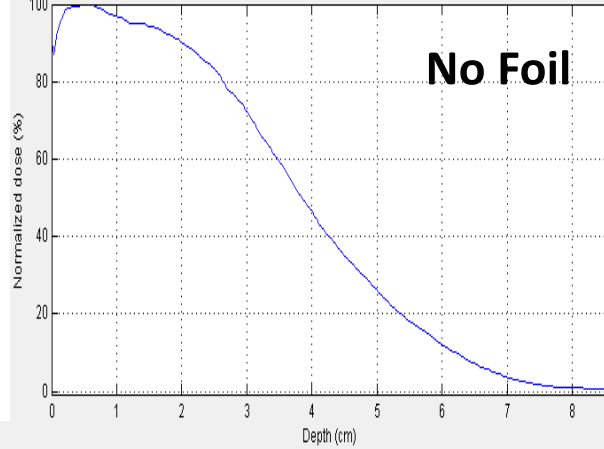

**(B)**

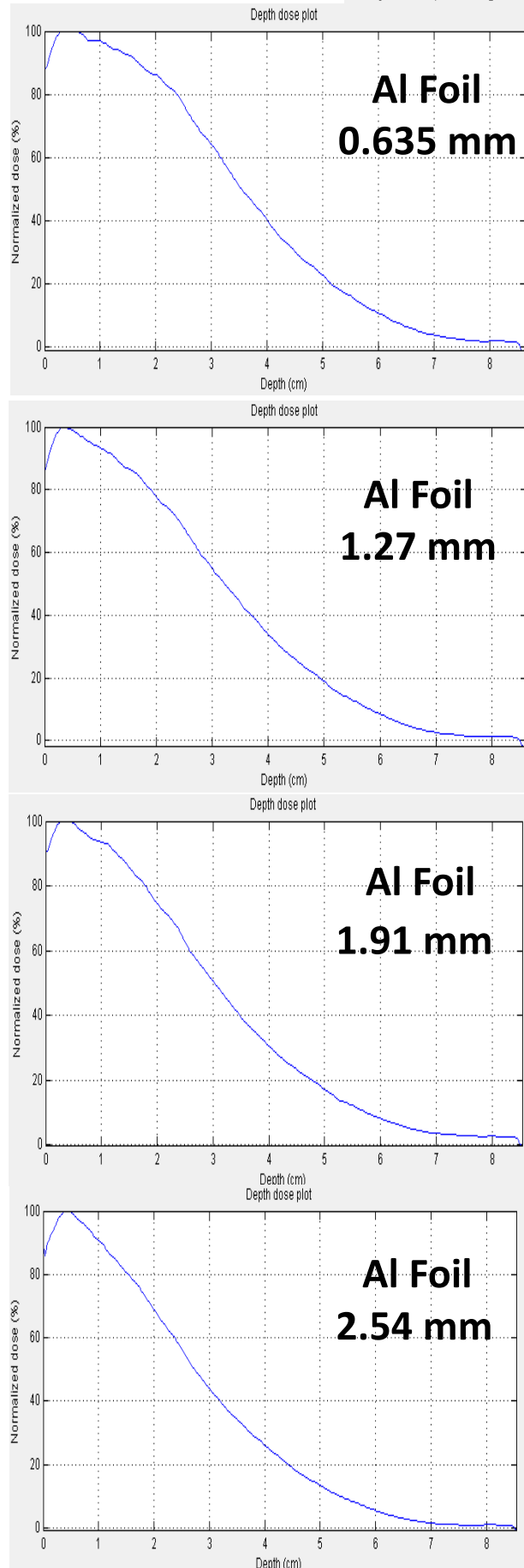

**(C)**

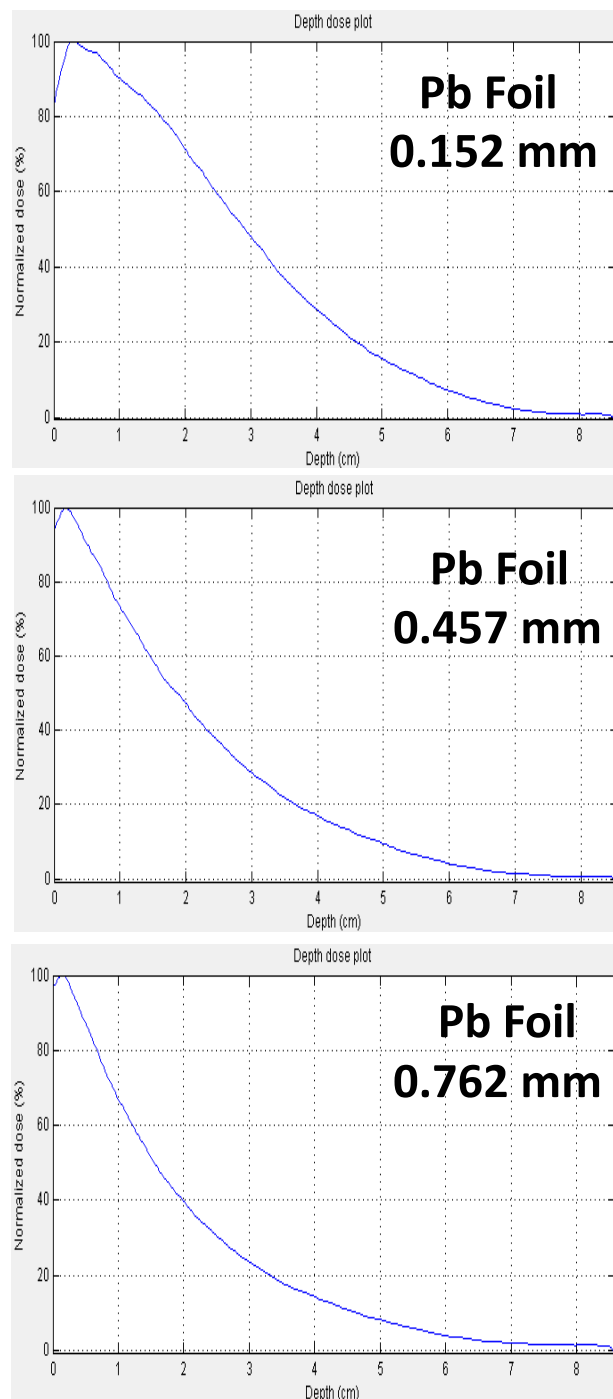

Increasing Foil Thickness

Supplement: Supplementary file 3 — Figure S3: PDDs for 15 MeV at 600 MUs. (A) no foil, (B) Al foil, (C) Pb foil. [file ACM2-27-e70484-s001.pdf]

(A)

Penumbra Size at  $D_{90}$  (cm)

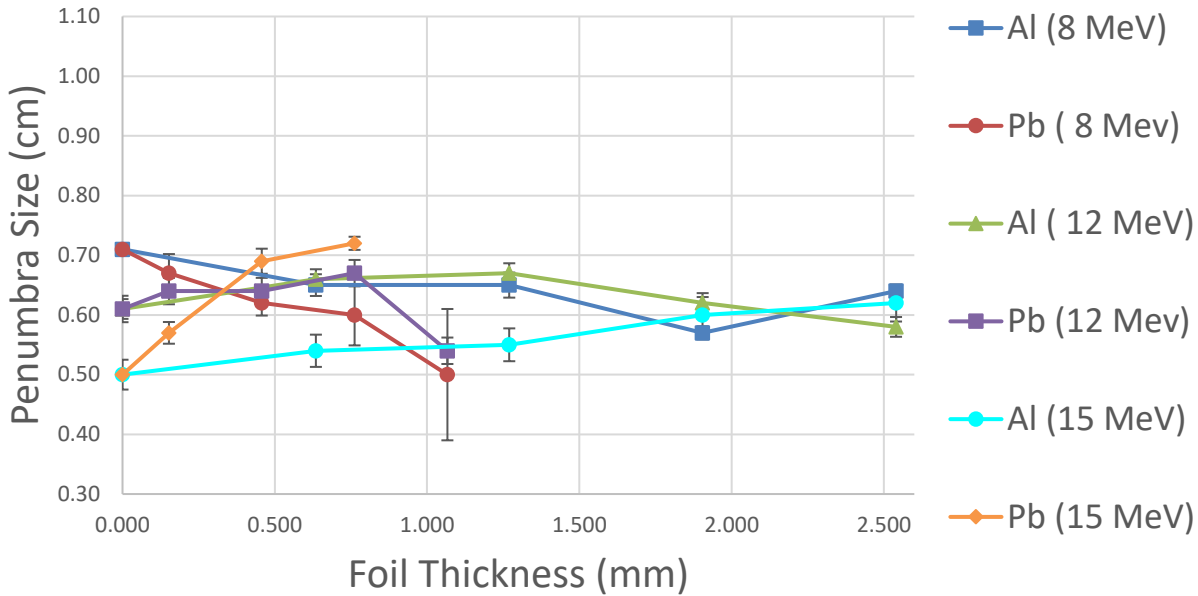

(B)

Penumbra Size at  $D_{50}$  (cm)

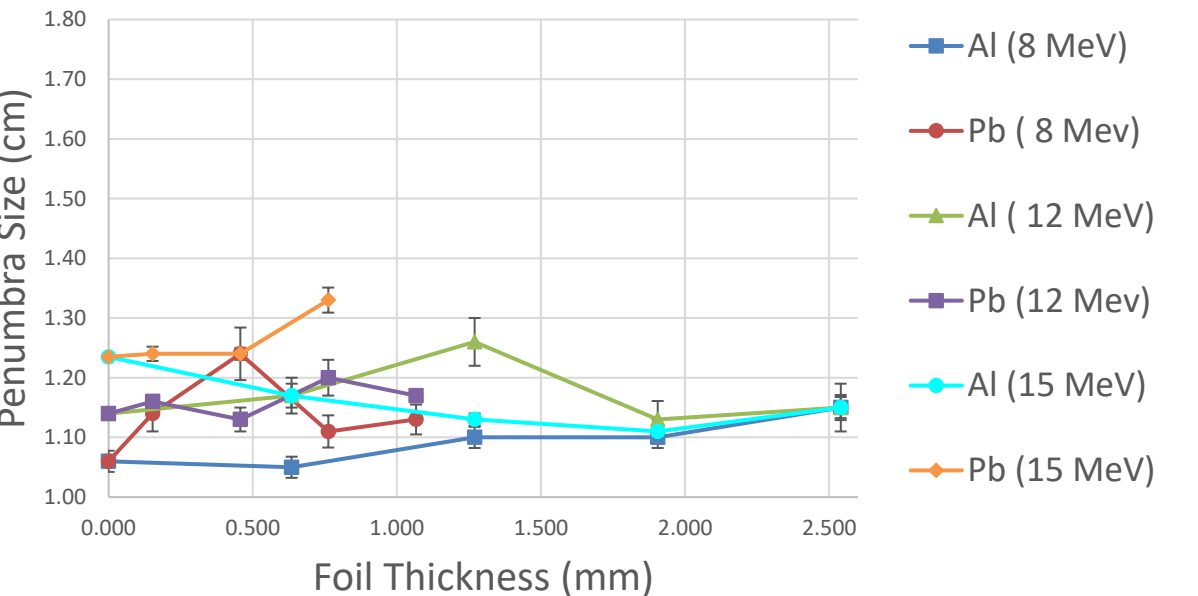

(C)

Penumbra Size at  $D_{25}$  (cm)

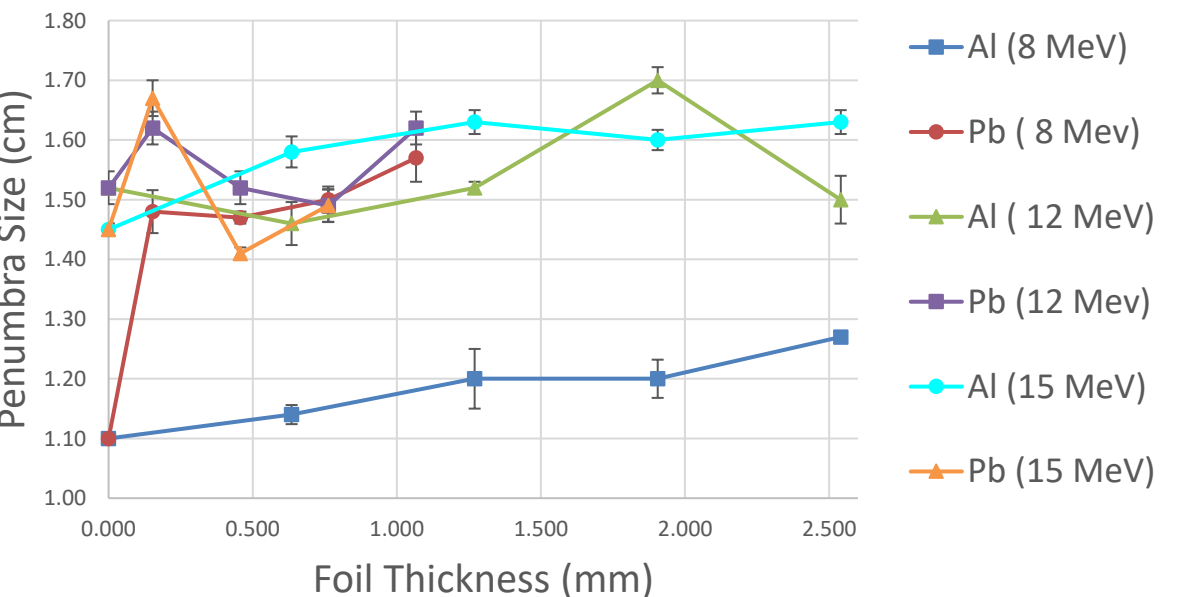

Supplement: Supplementary file 4 — Figure S4: Penumbra size at (A) D90 (cm), (B) D50 (cm), (C) D25 (cm) of Pb & Al scatter foils for 8, 12 & 15 MeV electron beams at 600 MUs. [file ACM2-27-e70484-s004.pdf]
